# A Tale of Tail Loss: Fine‐Scale Landscape Composition Predicts Caudal Autotomy Along an Urban Gradient in Two Congeneric Lizards

**DOI:** 10.1002/ece3.73906

**Published:** 2026-06-28

**Authors:** Natan Gottari, Mattia Falaschi, Stéphanie Sherpa, Agostino Apa, Giulia Arioli, Klevisa Bezhani, Bruno D'Adda, Gentile Francesco Ficetola, Chiara Marialuisa Manzo, Francesco Rosadini, Andrea Melotto

**Affiliations:** ^1^ Department of Biotechnologies e Biosciences Università Degli Studi di Milano‐Bicocca Milan Italy; ^2^ Department of Environmental Science and Policy Università Degli Studi di Milano Milan Italy; ^3^ Department of Life and Environmental Sciences (DISVA) Università Politecnica Delle Marche Ancona Italy; ^4^ Department of Earth Sciences Sapienza Università di Roma Rome Italy; ^5^ University Grenoble Alpes, University Savoie Mont Blanc, CNRS, LECA Grenoble France; ^6^ Department of Animal Ecology, Ecology Building Lund University Lund Sweden; ^7^ Max‐Planck Institute of Animal Behavior Radolfzell Germany

**Keywords:** landscape composition, *Podarcis* lizards, predator–prey interactions, spatial scale, tail regeneration, urban ecology

## Abstract

Human‐modified landscapes alter species interactions, yet the spatial scale at which landscape features influence predator–prey interactions remains poorly understood. Caudal autotomy, a widespread antipredator defense in many reptile species, has been proposed as a proxy of predation pressure. We investigated landscape‐scale variation in tail autotomy frequency in two synanthropic lizards, *Podarcis muralis* and *P. siculus* , across four metropolitan areas in Italy. We combined extensive field sampling (386 individuals) across 33 localities along a gradient of urbanization with multi‐scale analyses of landscape composition and configuration, as well as proxies of predator occurrence (citizen‐science records, species distribution models, and the distribution of colonies of free‐ranging cats). Using generalized linear mixed models, we assessed which landscape features and spatial scales best explained variation in autotomy frequency. Overall, 66% of individuals exhibited autotomy. The frequency of autotomy was higher in *P. muralis* than in *P. siculus* and increased significantly with body size. Landscape composition strongly influenced the frequency of autotomy. Forest cover within 50 m of capture sites was the strongest predictor of autotomy, with individuals from areas characterized by lower forest cover showing a higher probability of tail loss. The explanatory power of landscape composition decreased at broader spatial scales. Instead, neither landscape configuration metrics, estimated predator richness, nor proximity to cat colonies significantly improved model performance. The frequency of caudal autotomy in *Podarcis* lizards varies along the urbanization gradient and is primarily associated with fine‐scale habitat structure. These findings underscore the strong role of landscape composition in shaping multiple animal traits and support the use of morphological indicators to assess the ecological effects of human‐driven landscape change.

## Graphical abstract

Human‐modified landscapes can alter predator–prey interactions, but the spatial scale at which landscape features influence these interactions remains unclear. We examined variation in tail autotomy, a proxy for predation pressure, in two synanthropic lizards across urban gradients. Using field data from four metropolitan areas in Italy, paired with multi‐scale landscape analyses, we showed that autotomy frequency was negatively associated with forest cover within 50 m, highlighting the importance of fine‐scale landscape composition in shaping predator–prey interactions.

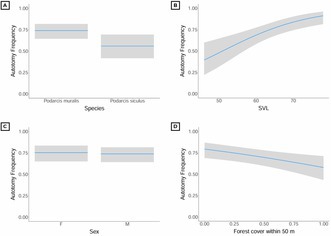

## 1 Introduction

Human modifications of landscapes have complex and multifaceted effects on biodiversity. The consequences of landscape modifications are manifold, including habitat loss, increased habitat fragmentation, changes in selective pressures and alterations of species interactions (Johnson and Munshi‐South 2017; Liu et al. 2016; Moreno‐García et al. 2025). These effects can also influence prey–predator interactions, with consequences that can scale from individual fitness to community composition and dynamics (Crooks and Soulé 1999; Fischer et al. 2012; Gilman et al. 2010; Tylianakis et al. 2008). Indeed, in human‐modified landscapes, local biota is known to change substantially (Aronson et al. 2014; Müller et al. 2013), often showing an increase in the frequency of alien or human‐associated species, including predators, with strong consequences for native prey species (Aronson et al. 2015; Chace and Walsh 2006; Crooks and Soulé 1999; Pyšek et al. 2010; Soulé et al. 1988). Besides, landscape modifications can affect species at multiple scales. Several studies have investigated the spatial scale at which habitat availability influences species, revealing complex patterns related to distribution, movement and to other processes occurring in the landscape, such as reproduction, foraging, and dispersal (Fahrig 2003; Ficetola et al. 2009; Turner 1989; Wiens 1989). For instance, the occurrence of several amphibians is positively associated with the cover of suitable habitats within 200–500 m, while the negative effects of roads can be detected over broad scales (1000–10,000 m), suggesting that the impacts of landscape alteration on dispersal act over broader scale compared to patch suitability (Ficetola et al. 2009; Marsh et al. 2017).

The issue of spatial scale can also apply to predation pressure (Wiens 1989). Human activities determine strong spatial variation in the distribution of predators at different trophic levels, with cascade effects on food webs potentially leading to the local declines of prey species (Crooks and Soulé 1999; Gregr et al. 2020). Assessing spatial variation in predation pressure is thus important to understand the consequences of landscape modifications on interspecific interactions. However, assessing the variation of predation pressure across human‐modified landscapes is challenging. Heterogeneity in environmental conditions can cause the distribution of suitable habitats for natural and introduced predators to vary across space, which makes it difficult to estimate predation risk for prey and the spatial scale at which it operates (Eötvös et al. 2018; Morozov 2022; Sorace and Gustin 2009). Accordingly, high‐resolution data on predator abundance and distribution are often lacking, and when available, are typically restricted to a limited number of taxa, small extent, or indirect estimates (Eötvös et al. 2018; Simon and Cresswell 2025). Under these circumstances, landscape‐level analyses can provide useful information to assess how predation risk can affect prey in human‐modified contexts.

Some lizard species are highly synanthropic and therefore represent valuable prey models to investigate prey–predator interactions in human‐modified landscapes (Pellitteri‐Rosa et al. 2017). Caudal autotomy, i.e., the voluntary detachment of the tail followed by regrowth, is a common defensive strategy in lizards. Caudal autotomy is primarily employed as an anti‐predator mechanism (Arnold 1984; Bateman and Fleming 2009; Bateman and Fleming 2011; Pafilis, Foufopoulos, et al. 2009), even if it can be influenced by additional factors and in some cases it has been interpreted as a result of intraspecific aggression (e.g., in insular systems, see Itescu et al. 2017). Tail loss can enhance survival by allowing individuals either to escape from a predator's grasp or diverting the predator's attention through the autonomous movements of the detached tail portion (Arnold 1988; Edmunds 1974; Pafilis et al. 2005). However, tail regeneration is associated with substantial costs. Regeneration requires a high energetic investment (Cooper et al. 2004; Maginnis 2006; Vitt et al. 1977) and tail loss can impair several physiological functions, as well as locomotor performance and social interactions (Ballinger and Tinkle 1979; Brown et al. 1995; Deimezis‐Tsikoutas et al. 2025; Downes and Shine 2001; Martin and Avery 1998; Smith 1996). Moreover, the regenerated portion of the tail consists of a continuous cartilaginous rod that replaces the original vertebrae; this structure lacks autotomy planes, thereby reducing the likelihood of subsequent autotomy events during future predatory encounters (Barr et al. 2019). Thus, tail autotomy confers immediate survival benefits yet entails trade‐offs that may compromise future individual fitness.

Empirically, the frequency of caudal autotomy varies substantially among lizard populations inhabiting environments characterized by different levels of urbanization (Tyler et al. 2016), possibly because human‐modified landscapes are characterized by distinct predator communities dominated by synanthropic species and/or domestic carnivores (e.g., cats, Ditchkoff et al. 2006), which are largely generalist predators. This lack of specialization may reduce predation efficiency and thus increase the probability of unsuccessful predation attempts, potentially leading to higher rates of autotomy (Medel et al. 1988). Therefore, spatial variation of caudal autotomy can provide an indirect but readily‐available measure of variation in risk exposure and predation pressure across landscapes that is otherwise difficult to achieve (Tyler et al. 2016), potentially helping to assess at which scale landscape modifications influence the intensity of predator–prey interactions. However, few studies have used lizard caudal autotomy to measure the predation pressure along an urbanization gradient (Bateman and Fleming 2011; Chapple and Swain 2004; Tyler et al. 2016), and limited data are available on the relationships between the occurrence of caudal autotomy and landscape features at multiple scales.

To address this knowledge gap, we assessed the landscape‐scale variation of caudal autotomy in two lizard species, the common wall lizard *Podarcis muralis* and the Italian wall lizard *P. siculus* , in four metropolitan areas. Using extensive field data across areas comprising a broad range of landscape alteration, we aimed to: (i) test whether the occurrence of tail autotomy varies along the urbanization gradient, as previously proposed for other species; (ii) identify the landscape features most strongly related to the variation of tail autotomy, and at which spatial scale caudal autotomy of the two *Podarcis* species responds to landscape modification; (iii) assess whether the distribution of natural predators, or colonies of free‐ranging cats, which are abundant predators in human‐modified landscapes, can explain the variation of autotomy frequencies.

## 2 Methods

### 2.1 Study Species and Field Sites

**FIGURE 1:**
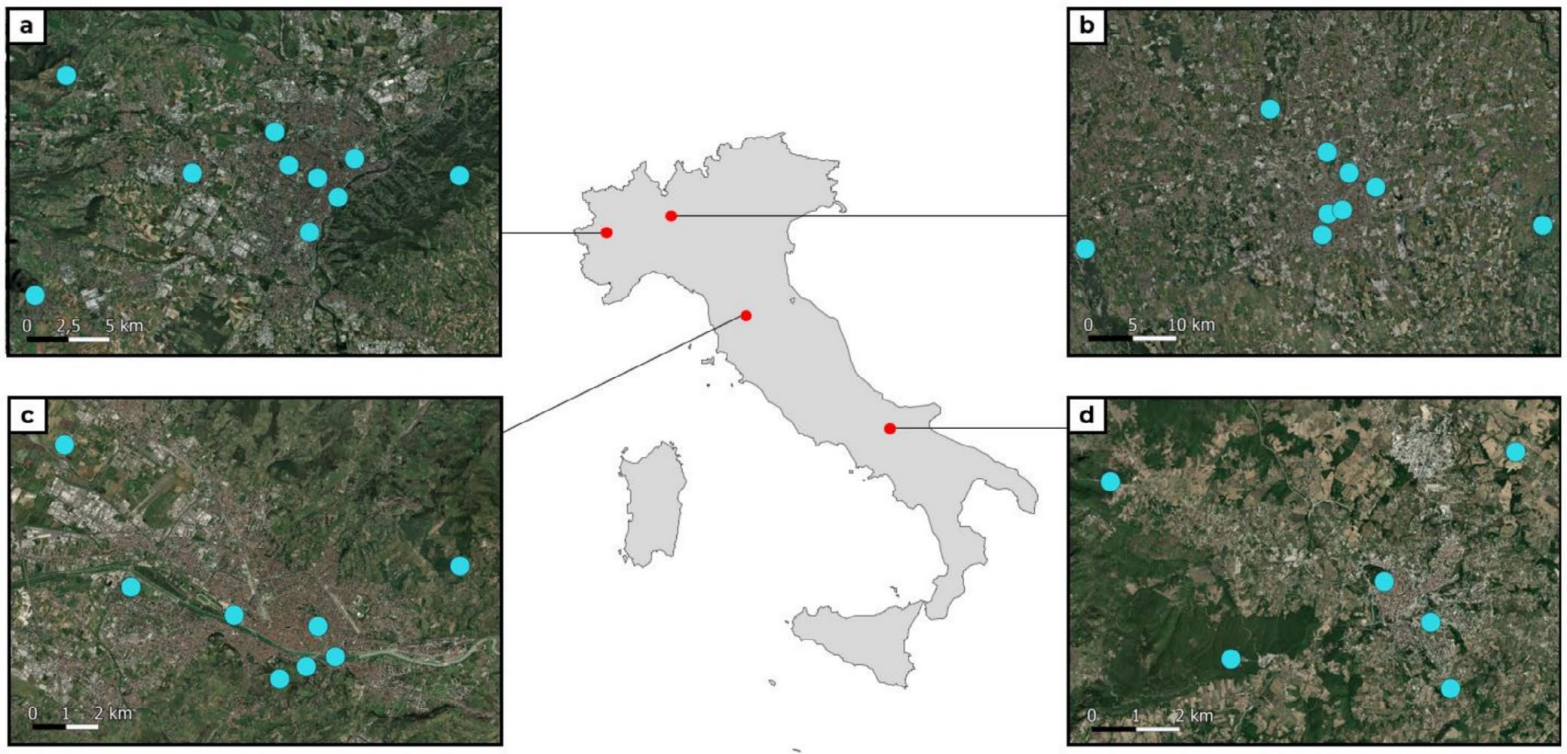
Study area and sampling sites. Location of the four study metropolitan areas in Italy: Turin (a), Milan (b), Florence (c), and Campobasso (d), and spatial distribution of the 33 sampling localities within each city (light blue points).

Our study focused on two *Podarcis* species: the Italian wall lizard, *Podarcis siculus*, and the common wall lizard, *P. muralis* . Both species are small lizards with a broad distribution in Southern Europe and inhabit a broad range of habitats, from seminatural to artificial landscapes. Both are highly synanthropic and widespread along the urbanization gradient (Arnold and Ovenden 2002; Biaggini et al. 2009; Böhme et al. 2009; Bowles 2024; Speybroeck et al. 2016). Between 2023 and 2025, we performed surveys within or nearby four metropolitan areas of Northern, Central and Southern Italy: Milan, Turin, Florence, and Campobasso. Within each metropolitan area, we selected 6–10 areas of ~1 km^2^ of surface (for a total of 33 areas, hereafter: localities), ranging from the city center to semi‐natural and natural areas outside cities (Figure 1). The distance between localities within each metropolitan area ranged from 1 to 52 km (average: 9.3 km). Distances between localities were well above the typical movement range reported for *Podarcis* species (home range ~50–400 m^2^; maximum distance between the edges of home ranges of adult individuals usually < 50 m) (Baldaccini et al. 2024; Biaggini et al. 2011; Foà et al. 1990; Mellado and Olmedo 1992; Scali et al. 2024), making the exchange or recapture of the same individuals between localities highly unlikely.

Each locality was surveyed in late spring (May–June), during daytime to match lizard activity period (approximately 5–hour survey per site), by teams of 3–5 operators. Lizards were captured using the noosing technique (Sutherland 2006). For each captured lizard, we recorded sex, snout‐vent length (SVL), and tail condition (intact = 0; regenerated or recently autotomized = 1). Tail regeneration was assessed by checking for changes in scale and color patterns that would indicate a discontinuity between the original tail and the point of autotomy (Seligmann et al. 2008). Very few individuals lost their tail during capture and were excluded from analyses. The exact coordinates of sampling points (i.e., the points where we captured each individual) were recorded with a GPS (accuracy: 3 m).

### 2.2 Landscape Composition and Configuration

We assessed landscape composition and configuration based on land cover maps retrieved from 2023 cover of forest and grassland at a very high resolution of 10 m (Copernicus Land Monitoring Service 2024a, 2024b). As a measure of the composition of landscape surrounding the sampling points, we calculated the percentage of grassland and forest in concentric buffers around each point (20 buffers with radii from 50 to 1000 m by steps of 50 m).

Furthermore, as a measure of landscape configuration, we calculated the following five metrics using the *landscapemetrics* package in R (Hesselbarth et al. 2019): number of patches (*lsm_c_np*), mean patch area (*lsm_c_area_mn*), effective mesh size (*lsm_c_mesh*), edge density (*lsm_c_ed*), and mean Euclidean nearest‐neighbor distance (*lsm_c_enn_mn*). These metrics describe different aspects of habitat spatial configuration, including the degree of fragmentation (number of patches), the average size of habitat patches (mean patch area), overall landscape connectivity (effective mesh size), the amount of habitat edge per unit area (edge density), and the spatial isolation of habitat patches (mean nearest‐neighbor distance). Landscape‐configuration metrics were calculated for forest cover within a 50 m radius around each sampling point. We focused on forest cover within 50 m because multi‐scale analyses (see Results) showed that forest cover within this radius is the land cover feature most strongly related to the occurrence of autotomy in the study species. When calculating edge density and mean nearest‐neighbor distance, we obtained a high number of missing values, likely due to the limited extent or absence of forest patches. Therefore, these variables were excluded from the analyses.

### 2.3 Estimating the Occurrence of Natural Predators

Given that predators are major drivers of caudal autotomy (Bateman and Fleming 2011), we developed a set of variables to consider the potential effects of predator occurrence. We obtained the list of the vertebrates that can prey on the two lizard species in Italy from Maiorano et al. (2020). Due to the lack of exhaustive high‐resolution data on the occurrence of predators, we estimated the presence of natural predators nearby sampling points using two alternative approaches. We acknowledge that information on predators was at a scale much coarser than some landscape features. Unfortunately, most predator data were only available at a 1‐km spatial resolution thus analyses at finer scales were not possible. We also highlight that many predators (e.g., birds, mammals) have rather large home ranges, thus they can exert predation pressures over broader scales than habitat distribution.

First, we extracted all records of occurrence of predators in areas within a buffer of 1 km around each sampling point from the Global Biodiversity Information Facility citizen science platform (GBIF; www.gbif.org), for the period 2005–2025 (assessment date: January 25, 2026). Second, as citizen science data are far from complete, we relied on the potential presence of predators, estimated on the basis of species distribution models of European vertebrates by Si‐Moussi and Thuiller (2024). These authors developed 1‐km resolution maps of predicted presence‐absence for a large number of European species, including terrestrial vertebrates, using an ensemble of Species Distribution Models (SDMs). SDMs were built on the basis of presence‐only data from GBIF and multiple environmental variables including land‐use, climate, terrain, hydrography and soil (Si‐Moussi and Thuiller 2024). These SDMs showed excellent performance, with a median validation score (true skill statistics) > 0.85 for all the tetrapods analyzed. Despite multiple limitations, SDM outputs are an effective strategy to obtain otherwise lacking broad‐scale data on potential species distribution (Araújo et al. 2025; Domisch et al. 2019; Morán‐Ordóñez et al. 2025; Rondinini et al. 2006), and were therefore used to support the analysis based on GBIF data. For both approaches, we calculated the total number of predator species (predator richness) within 1 km. Preliminary analyses considering specific categories (e.g., mammals only and reptiles only) yielded nearly identical results.

### 2.4 Occurrence of Colonies of Free‐Ranging Cats

To account for possible predation due to cat presence, we obtained data on the presence and spatial location of cat colonies from the veterinary Local Health Authorities (“Aziende Sanitarie Locali”) associated with the municipalities where sampling took place, covering 24 municipalities in total. The spatial behavior of cats belonging to managed colonies is poorly documented, but previous studies suggest an average range of movements of 50–150 m for domestic cats, around 2 km for feral cats, and 80–150 m for farm cats (Barratt 1997; Edwards et al. 2001; Fardell et al. 2021; Kays et al. 2020; McGregor et al. 2015; Thomas et al. 2014). After gathering information about the cat colonies located nearby the sampling localities, we calculated three variables as potential measures of the impact of free‐ranging cats: the number of colonies within a buffer of 300 m or 500 m around sampling points and the distance from the nearest colony of each sampling point. The limited spatial accuracy of cat colony locations, with some records being associated with uncertainty up to ~100 m, hampered the inclusion of cat‐related variables at finer spatial scales.

### 2.5 Statistical Analyses

Initially, we used generalized linear mixed models (GLMMs) with a binomial error structure to assess whether species identity and individual‐related biological features (sex and SVL) affect the variation of the occurrence of tail autotomy. City was included as a random effect to account for the non‐independence of observations collected within the same spatial context. In lizards, size tends to increase with age, but this relationship is not necessarily linear (Zuffi et al. 2025). Nevertheless, preliminary analyses testing a quadratic term did not show significant deviations from the linear pattern; thus, in our models we assumed a linear relationship between SVL and autotomy frequency. GLMMs were fitted using the *lme4* package in R (Bates et al. 2015); the significance of terms was assessed using a likelihood ratio test.

We then adopted a multi‐scale approach to identify the land cover type and the spatial scale that best explained the variability observed among populations. We considered two land‐cover typologies: forest and grassland within 50, 100, 250, 500, and 1000 m from the sampling points. The support of the different land‐cover typologies and scales was assessed by adding each of them at each radius to the GLMM including species identity, sex and SVL. The support of the vegetation type and radius was assessed by calculating the reduction in the bias‐corrected Akaike's Information Criterion (AICc) compared to the models without landscape composition (Burnham and Anderson 2002). The collinearity between land cover typologies hampered the inclusion of both forest and grassland within the same model. In a second step, we refined this analysis by focusing on the land cover type receiving the strongest model support in the above‐described analysis. For this land cover type, the multi‐scale analysis was repeated with finer spatial scale intervals, considering all the 20 buffers from 50 to up to 1000 m. Again, the best supported scale was assessed on the basis of AICc reduction compared to the model without landscape composition. To take into account the uncertainty associated with AICc‐based model selection when a large number of variables are considered, we also calculated the AICc weights *w*, which represent the support of the model, given the data (Burnham and Anderson 2002).

Subsequently, we tested whether additional factors representing (1) landscape configuration or (2) the occurrence of wild predators and (3) cat colonies further explain the spatial variation of tail autotomy. First, we assessed the potential role of landscape configuration, beyond the effect of landscape composition (Fahrig 2003). Two of the three selected metrics of landscape configuration showed extremely high correlation with forest cover (effective mesh size: Pearson's *r* = 0.94; mean patch area: Pearson's *r* = 0.96). Therefore, these metrics were not included in the model to avoid multicollinearity, and because they do not represent a measure of configuration that is independent from the one of composition (Dormann et al. 2013; Fahrig 2003; Fahrig et al. 2026). Conversely, the number of patches was not correlated with forest cover (Pearson's *r* = 0.10). To evaluate the potential impact of landscape configuration, we thus added the number of patches to the best model including land cover and tested if it improves the performance (measured by AICc) of the model. Configuration was assessed within the same radius as land cover.

Second, to assess the potential role of natural predators, we added predator richness to the best AICc model previously identified. We repeated this analysis for the two approaches used to calulate predator richness (citizen science data and SDM‐output). Third, to assess the potential role of cat colonies, we included alternatively the log‐transformed distance from the nearest cat colony, or the number of colonies within 300 or 500 m to the best model considering landscape composition. The potential impact of predator‐associated metrics was evaluated by assessing whether each predictor added to the best landscape composition model improved model performance.

For predictors included in the final model, we also tested two‐way interactions with species identity to assess whether effects differed between the two species. Interaction terms were retained in the final model only if they showed significant effects.

We used two approaches to confirm that the results of the final model were not affected by the non‐independence of lizards captured in the same locality, or by spatial autocorrelation. First, we built a GLMM including locality as an additional random effect, and assessed the consistency with the model without locality. In this model, locality explained essentially no variation (variance component: 1.568 × 10^−10^), and the AICc was larger than that of the model without locality (model with locality: AICc = 479.23; model without locality: AICc = 477.15). Furthermore, all the effects were identical to those of the model without locality (Table S1), suggesting that the identity of locality does not need to be included as an additional random factor in the model (West et al. 2022).

Second, we built a GLMM including geographic coordinates of sampled individuals as a spatial error term, in order to confirm that our results are not biased by spatial autocorrelation between individuals from the same city. The model included the same predictors of the best model and was fit using penalized quasi‐likelihood (*glmmPQL* function from the R *MASS* package), also including city as a random effect to account for unmodelled city‐level variation (Breslow and Clayton 1993; Dormann et al. 2007; Venables and Ripley 2002). glmmPQL models provide excellent performance with spatially explicit data but cannot be used for model selection because they are estimated using penalized quasi‐likelihood; thus, AIC values are not available with this approach. However, consistency of coefficients between models including or not including spatial terms indicated that the results of the standard GLMM are not biased by autocorrelation (Beale et al. 2010; Dormann et al. 2007; Table S1). All the analyses were performed in the R environment (version 4.4.1), using packages *lme4*, *MuMIn*, *car*, *MASS*, and *nlme* for statistical modeling, while *visreg* and *ggplot2* were used to generate graphs showing model outcomes.

## 3 Results

### 3.1 Frequency of Autotomy and Individual‐Related Biological Features

Overall, we captured 386 individuals (278 *Podarcis muralis* , 108 *P. siculus* ), 66% of which exhibited a regenerated tail (Table S2). When we modeled the frequency of autotomy as a function of the biological features of individuals, we found a significantly lower frequency of autotomized tails in *P. siculus* compared to *P. muralis* ( *P. siculus* : 62.03%; *P. muralis* : 67.62%; *β* = −0.641; odds ratio, OR = 0.526; *χ* ^2^_1_ = 4.317; *p* = 0.038). Furthermore, the frequency of autotomy increased in individuals with longer snout‐vent length (*β* = 0.094; OR = 1.098 per mm of SVL; *χ* ^2^_1_ = 17.564; *p* < 0.001). No significant differences were detected between males and females (*β* = −0.125; OR = 0.882; *χ* ^2^_1_ = 0.289; *p* = 0.590).

### 3.2 Multi‐Scale Effects of Landscape Composition and Configuration

**FIGURE 2:**
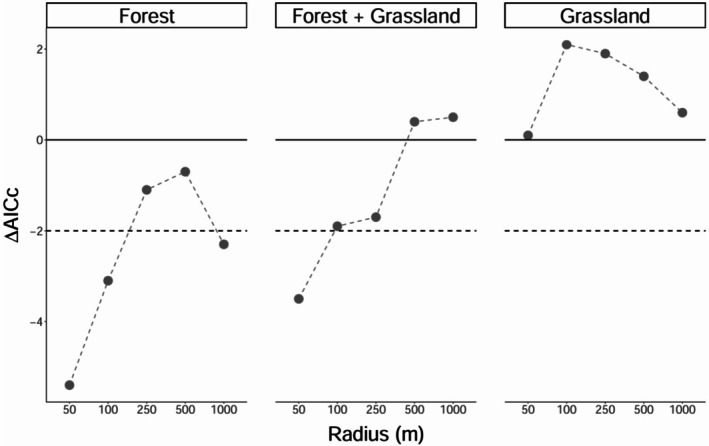
Importance of landscape composition on tail autotomy. Model support (assessed using ΔAICc) of models including forest cover, grassland cover, or both predictors simultaneously at multiple scales (see Methods). All models included the same set of covariates (species, SVL, sex) and only differed for the inclusion of landscape cover predictor or the spatial scale at which the predictor was quantified. ΔAICc represents the reduction in Akaike's Information Criterion relative to the model without landscape composition. Points are connected by a line for visual guidance. The solid line at ΔAICc = 0 represents the model without landscape composition, while the dashed line at ΔAICc = −2 indicates the threshold for substantial improvement.

Adding landscape composition (grassland and forest cover) to the model including species identity and the individual‐related biological features improved the fit of models (Table S3). The strongest reductions of AICc values were observed for models including forest cover, particularly at fine spatial scales, while models including grassland cover alone or in combination with forest did not substantially improve model performance (Figure 2 and Table S3).

**FIGURE 3:**
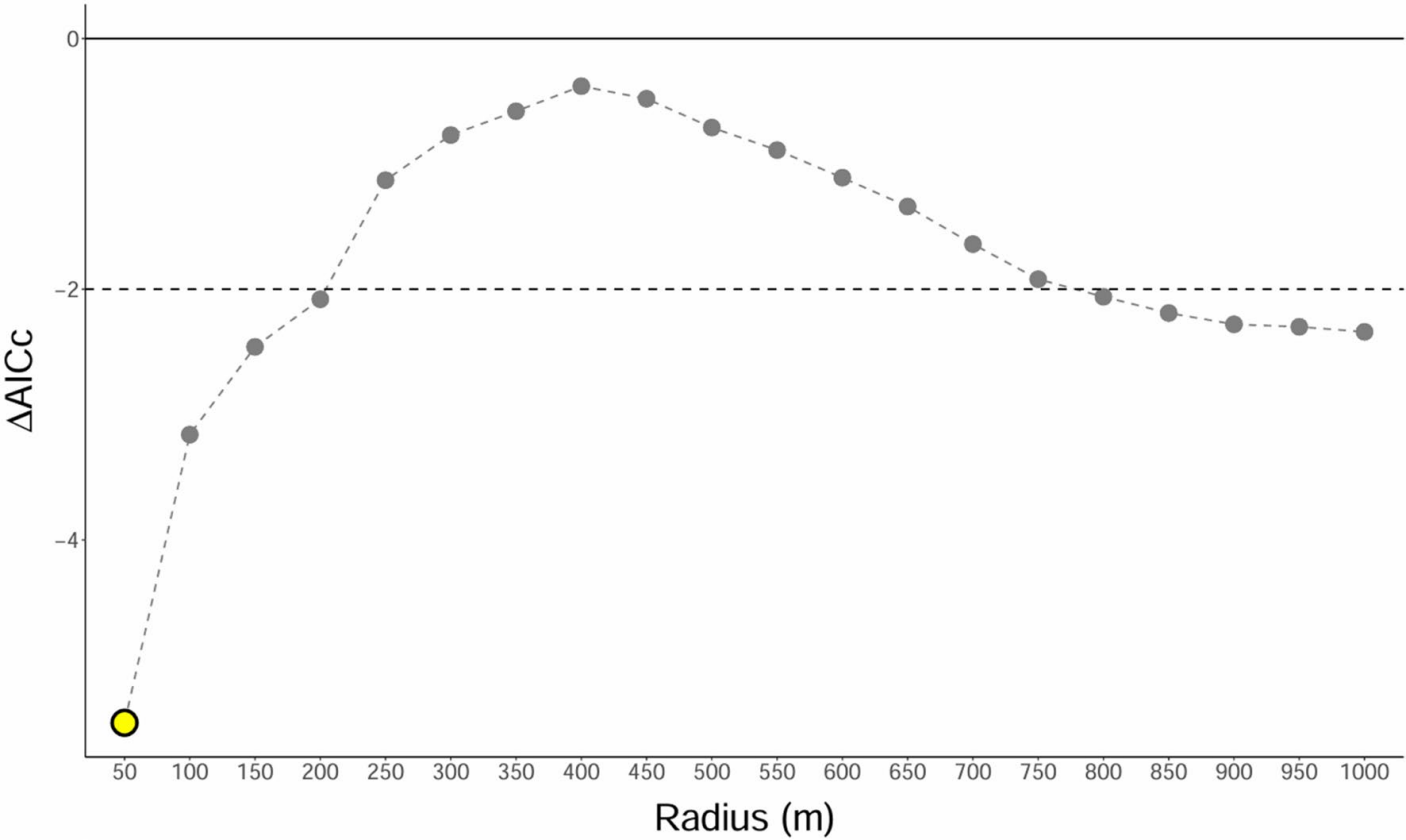
Scale‐dependent effect of forest cover on tail autotomy. Model support (ΔAICc) across spatial scales for forest cover measured within 20 buffers around the sampling sites, with radii ranging from 50 to 1000 m. ΔAICc represents the reduction in Akaike's Information Criterion relative to the model including species, sex and SVL (without landscape composition). Points are connected by a line for visual guidance. The solid line at ΔAICc = 0 represents the model without landscape composition, while the dashed line at ΔAICc = −2 indicates the threshold for substantial improvement. The yellow point indicates the spatial scale with the strongest support.

When we analyzed in detail the relationship between autotomy frequency and forest cover, we observed a clear variation of the effect of forest cover across spatial scales. The frequency of autotomy was best explained by forest cover within 50 m of the sampling points, while the importance of forest cover tended to decrease when measured over broader buffers (Figure 3). Forest cover within 50 m of sampling points ranged from 0% to 100% (mean: 37%). The model with forest cover within 50 m also showed the highest AICc weight (*w* = 0.235). Only three models showed AICc weight > 0.05, and they included forest cover within 50 (*w* = 0.235), 100 (*w* = 0.074) or 150 m (*w* = 0.052) as landscape predictors (Table S3). This suggests that, despite some uncertainty in model selection, variation of autotomy is related to landscape variation at fine spatial scale. Note that forest cover showed a strong, negative correlation with urban cover (Spearman's correlation: *r* = −0.42, *p* < 0.0001; analysis performed at the 50 m scale), thus low values of forest cover are generally associated with human‐dominated landscapes within urban areas.

**FIGURE 4:**
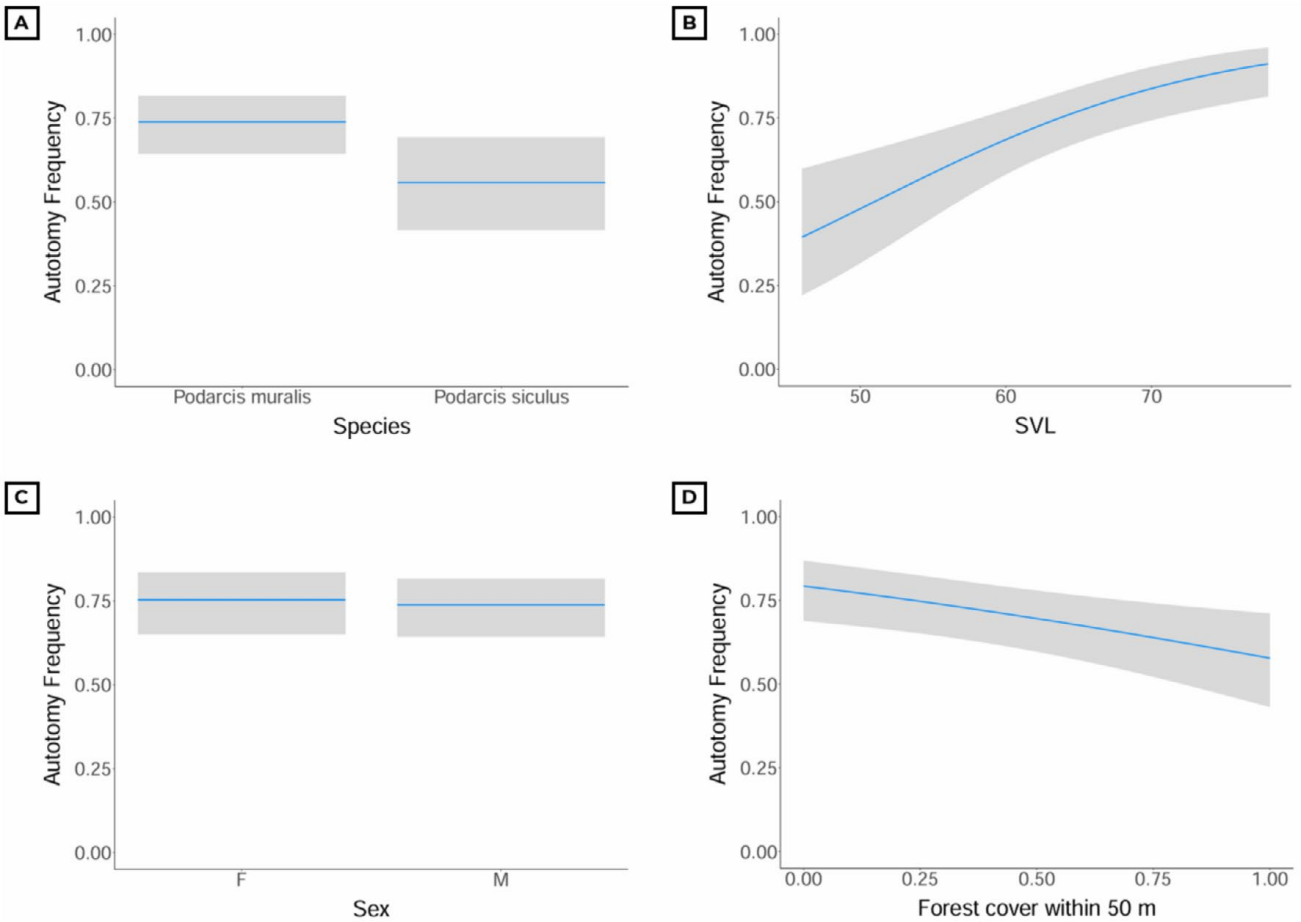
Results of the GLMM showing the effects of the selected predictors on caudal autotomy: (A) species (*p* = 0.015), (B) SVL (*p* < 0.001), (C) sex (*p* = 0.741), and (D) forest cover within 50 m (*p* = 0.006). Blue lines represent model predictions and gray shaded areas indicate 95% confidence intervals.

Overall, the model including species identity, the individual features and forest cover within 50 m showed a significant decrease of the probability of observing autotomized individuals in areas with high forest cover (*β* = −1.027; OR = 0.36; *χ* ^2^_1_ = 7.41; *p* = 0.006), and confirmed the higher frequency in *P. muralis* and in individuals with longer SVL (Table 1 and Figure 4). None of the tested two‐way interactions between predictors and species identity was significant (all *p* > 0.2), indicating that the effects of sex, body size and forest cover were consistent between species.

**TABLE 1:** Summary of the generalized linear mixed‐effects model with lowest AIC, assessing the effects of sex, body size (SVL), species, and forest cover within 50 m of the sampling points on the probability of caudal autotomy. Odds ratios (OR) are calculated as the exponential of *β*.

Fixed effect	*β*	SE	OR	*χ* ^2^_1_	*p*
Species: *P. siculus* (vs *P. muralis* )	−0.806	0.331	0.447	5.915	**0.015**
SVL	0.086	0.023	1.090	14.259	**< 0.001**
Sex	−0.078	0.237	0.925	0.109	0.741
Forest cover 50 m	−1.027	0.377	0.358	7.417	**0.006**

*Note:* Significant relationships are in bold.

Adding landscape configuration (number of patches) to the model including forest cover within 50 m did not improve model performance (Table S4).

### 3.3 Potential Effects of Natural Predators

Based on the database of trophic web interactions (Maiorano et al. 2020), 100 vertebrate predator species potentially preying on *P. muralis* and *P. siculus* occur in Italy (Table S5). Based on GBIF occurrence records, 47 out of these predators were identified within a buffer of 1 km from the sampling points (Table S5). However, including the total number of detected predator species within 1 km did not significantly improve the performance of models (Table S4). Species distribution models suggested the possible occurrence of 82 predator species in the cells where we captured lizards (Table S5). However, also in this case, including the number of predator species did not improve models (Table S4).

### 3.4 Potential Effects of Colony Cats

Overall, we identified 524 cat colonies nearby the sampling localities. A linear model showed that the distance between each sampling point and the nearest colony was significantly related to landscape composition, as the sites with highest forest cover within 50 m were the furthest ones from the nearest cat colony (*β* = 902 ± 238 m, *t* = 3.78, *p* < 0.001; *R* ^2^ = 0.033). Nevertheless, including the distance from the nearest cat colony or the number of colonies within a buffer of 300 or 500 m around sampling points did not improve model fit (Table S4).

## 4 Discussion

The frequency of tail autotomy in *Podarcis* lizards showed strong variation across landscapes with different composition. Lizards inhabiting human‐dominated landscapes exhibited more frequent autotomy compared to individuals living in environments with more natural vegetation, in agreement with previous observations in other lizard species (Tyler et al. 2016). After accounting for landscape composition, landscape configuration or the estimated presence of predators seems to play a limited role, highlighting the key role of landscape composition as a main predictor of multiple performance‐related traits (Fahrig 2003).

Testing the impact of landscape composition using multiple concentric buffers showed that the frequency of tail autotomy was most strongly related to forest cover measured within a 50 m radius around sampling points (Figure 4D). Multi‐scale analyses are a powerful approach to assess at which grain environmental modifications influence species and relate them to specific ecological processes (Ficetola et al. 2004; Jackson and Fahrig 2015; Pellet et al. 2004). The fine spatial scale identified here aligns with the small home ranges typical of *Podarcis* lizards. Previous studies report adult home ranges generally spanning 50–400 m^2^ in these species, corresponding to spatial extents on the order of tens of meters, with males typically occupying larger areas than females (Baldaccini et al. 2024; Biaggini et al. 2011; Foà et al. 1990; Mellado and Olmedo 1992; Scali et al. 2024). Even if juveniles can disperse over longer distances, the movements of these lizards usually are limited, suggesting that most ecological interactions, including predator encounters, occur at very fine scales (Mellado and Olmedo 1992; Scali et al. 2024; Vignoli et al. 2012; Vignoli et al. 2015). Consequently, habitat features measured within a few tens of meters are likely to reflect the conditions experienced by individuals more accurately than broader‐scale metrics.

Identifying the processes explaining variation of caudal autotomy can be complex, as many drivers can contribute to shape its frequency under different environmental contexts. First, the integration of our results with previous data on urban lizards suggests a role of pressure by low‐efficiency predators (i.e., predators for whom predation attempts more often result in caudal autotomy than capture; Schoener 1979). In fact, several authors have proposed that higher autotomy frequencies in human‐dominated landscapes are related to the greater presence of inefficient predators (Bateman and Fleming 2011; Medel et al. 1988; Tyler et al. 2016). Among them, domestic cats ( *Felis catus* ) often capture lizards and are particularly abundant in urban areas (Bateman and Fleming 2011; Chapple and Swain 2004; Koenig et al. 2002; Mori et al. 2019). Well‐fed domestic or colony cats tend to play with their prey rather than consume it immediately. As a result, they may generate more non‐lethal predatory encounters, potentially increasing the observed frequency of tail autotomy (Bateman and Fleming 2011; Chapple and Swain 2004; Leyhausen and Tonkin 1979; Morris 1986). In contrast, natural predators are typically more efficient, as they often kill their prey during attacks, leaving fewer opportunities for autotomy (Blomberg and Shine 2000; Chapple and Swain 2004; Medel et al. 1988). However, we only found indirect evidence of the possible effect of cats, as neither cat colonies nor wild predators explained the frequency of autotomy beyond the effects of landscape cover. This likely occurred because data on the actual presence of predators are limited, and we relied on proxies that provide an imperfect representation of their impact, complicating a thorough assessment of their effects. Despite these limitations, our data confirmed the relationship between landscape features and predator distribution, as colonies generally were distant from forested areas, supporting the hypothesis that the spatial variation of these human‐associated predators can contribute to the observed pattern of autotomy.

Second, predator exposure can be affected by the variation in habitat structure and complexity, due to different refuge availability. The availability of refuges can strongly vary along the urbanization gradient (Batabyal et al. 2017; Brock and Bednekoff 2025), being typically more abundant under natural environments, where vegetation can offer more shelter against predators. The increased abundance of complex microhabitats, limiting exposure to predators or facilitating escape response, can play a role in explaining the lower frequency of caudal autotomy in natural landscapes (Martin and López 1995; Schooley et al. 1996). Conversely, the reduced shelter availability in human‐dominated landscapes, together with a higher prevalence of inefficient human‐associated predators, may induce higher tail autotomy rates. Third, boldness and risk‐taking behavior are often more pronounced in animals inhabiting urban landscapes (Sol et al. 2013), including the two studied lizards (Brock and Bednekoff 2025; Pellitteri‐Rosa et al. 2017), and this can increase their exposure to predators. Altogether, these factors can contribute to explaining the observed pattern of enhanced autotomy frequency with declining forest cover. Finally, it is also possible that other processes, unrelated to predators, contribute to the spatial variation of autotomy. For instance, on islands, intraspecific interactions in high‐density populations may lead to a higher autotomy frequency (Itescu et al. 2017). Habitat features, such as the cover of urban vs. natural habitat, can influence population density, potentially intensifying the competition for space and food, increasing intraspecific aggression, and possibly affecting the rate of tail loss (Nunes and Carretero 2024; but see Maune et al. 2025). Still, more data are required to test these hypotheses.

In our study, natural predators were assessed using citizen‐science data and species distribution models. Both approaches are boosting our knowledge of biodiversity patterns, also in urban areas, because they enable obtaining fine‐scale distribution data in the absence of intensive monitoring programs (Acevedo‐Charry et al. 2025; Chapman et al. 2024; Crawford et al. 2020; Guisan and Thuiller 2005; Row et al. 2025; Zimmermann et al. 2010), but they also have their own limitations. Citizen science records represent only a fraction of true predator presence and tend to overrepresent species frequenting human‐dense areas (e.g., Boakes et al. 2016). Species distribution models provide maps of suitability, which sometimes can overestimate the actual presence of species, have a resolution that is coarser than the one of habitat data, and offer imperfect predictions that are, for instance, affected by the quality of calibration records and the selection of predictors (Beck et al. 2014; Bracho‐Estévanez et al. 2024; Cánibe et al. 2022; Guisan and Rahbek 2011; Habel et al. 2025; Kramer‐Schadt et al. 2013). Similarly, data on cat colonies represent the only consistent metric on cat distribution currently available at the municipality level, but were probably not exhaustive, and cat colonies only comprise part of cats inhabiting human‐dominated areas. Moreover, the limited spatial accuracy of colony locations hampered the possibility of analyses at very fine spatial scales. Therefore, both species distribution models and citizen science data must be interpreted with care, as they may be affected by multiple sources of uncertainty. In the future, a more accurate assessment of the abundance of both natural and domestic predators would be important to understand the actual variation of these key components of the ecological community inhabiting human‐dominated landscapes; for instance, this could be achieved through the implementation of repeated, targeted monitoring protocols (Mori et al. 2019; Wintle et al. 2010; Yoccoz et al. 2001). Moreover, quantifying the predatory efficiency of each species would be important to evaluate its actual contribution to lizard autotomy. Although some attempts have been made in this direction, we are still far from a comprehensive understanding (Chapple and Swain 2004; Medel et al. 1988).

At the individual level, the probability of tail autotomy increased significantly with body size (SVL), which, in lizards, tends to increase with age. This pattern is consistent with most previous studies (Brown and Ruby 1977; Chapple and Swain 2004; Turner et al. 1982; Wilson and Booth 1998) and has been related to the growing cumulative probability of encountering a predator over a lifetime, and to the higher visibility and conspicuousness for predators of larger individuals (Bateman and Fleming 2011; Chapple and Swain 2004; Tyler et al. 2016). Nevertheless, some studies suggested that higher autotomy in juveniles can occur, especially in populations with high lizard densities, potentially due to behavioral mechanisms forcing them into suboptimal habitats with fewer refuges as a result of exclusion by adults (Brandl and Völkl 1988) or because of an increase in intraspecific agonistic interactions, including adult‐juvenile aggressions (Cooper Jr et al. 2015; Pafilis, Meiri, et al. 2009). In our study, the frequency of autotomy did not show differences between males and females. While previous research suggested that, in some conditions, intraspecific interactions can drive autotomy (Itescu et al. 2017), this is generally accompanied by higher autotomy frequency in males, who are more aggressive towards male conspecifics (Donihue et al. 2016; Ficetola et al. 2024; Itescu et al. 2017). The lack of differences in autotomy between sexes (Table 1) suggests that sex‐related processes, such as intraspecific interactions, do not play the strongest role in determining the observed spatial variation. Moreover, recent findings suggest that in urban landscapes, where individuals are typically exposed to higher conspecific densities, aggressive interactions may be damped (Maune et al. 2025), further suggesting intraspecific interactions may not be the primary driver of the observed variation in tail loss.

Finally, the lower frequency of autotomy in *P. siculus* may reflect species‐specific differences in habitat use, activity and behavior, or antipredator strategies. Despite both lizards being generalists and highly synanthropic, in urban environments *P. siculus* is often found in microhabitats with abundant vegetation that can provide shelter, while *P. muralis* tends to prefer exposed walls with limited vegetation or crevices, where predation pressure can be particularly high (Rugiero et al. 2021; Simbula et al. 2019). Furthermore, in our dataset *P. muralis* was associated with the largest cities (Milan and Turin) where the frequency of non‐natural or inefficient predators might be particularly high. The two species might also differ in intrinsic easiness or propensity to perform tail‐shedding, a trait known to vary among lizard species and populations and which can depend on both anatomical and behavioral features (Pérez‐Mellado et al. 1997). Further studies are required to investigate the mechanisms underlying inter‐specific differences.

Overall, our study demonstrates that caudal autotomy frequency in *Podarcis* lizards varies along the urbanization gradient and is strongly associated with local landscape composition, particularly forest cover at very fine spatial scales. This highlights the pervasive impact of landscape composition on multiple traits of animal populations and pinpoints the usefulness of morphological features to measure the complex consequences of human modifications of landscapes on animal populations. Future studies based on direct and standardized monitoring of both wild and domestic predators will be crucial to clarify the mechanisms driving autotomy frequency in human‐modified landscapes.

## Author Contributions

**Natan Gottari:** conceptualization (equal), formal analysis (lead), investigation (equal), methodology (equal), writing – original draft (lead). **Mattia Falaschi:** conceptualization (supporting), formal analysis (equal), investigation (supporting), methodology (equal), writing – review and editing (equal). **Stéphanie Sherpa:** investigation (equal), project administration (equal), writing – review and editing (equal). **Agostino Apa:** investigation (equal), writing – review and editing (supporting). **Giulia Arioli:** investigation (equal), writing – review and editing (supporting). **Klevisa Bezhani:** investigation (equal), writing – review and editing (supporting). **Bruno D'Adda:** investigation (equal), writing – review and editing (supporting). **Gentile Francesco Ficetola:** conceptualization (equal), formal analysis (equal), funding acquisition (lead), methodology (equal), writing – original draft (equal). **Chiara Marialuisa Manzo:** investigation (equal), writing – review and editing (supporting). **Francesco Rosadini:** investigation (equal), writing – review and editing (supporting). **Andrea Melotto:** conceptualization (equal), formal analysis (supporting), investigation (equal), methodology (equal), supervision (lead), writing – original draft (equal).

## Funding

Project funded under the National Recovery and Resilience Plan (NRRP), Mission 4 Component 2 Investment 1.4—Call for tender No. 3138 of 16 December 2021, rectified by Decree n.3175 of 18 December 2021 of the Italian Ministry of University and Research funded by the European Union—NextGenerationEU, Project code CN_00000033, Concession Decree No. 1034 of 17 June 2022 adopted by the Italian Ministry of University and Research, CUP, H43C22000530001 Project title “National Biodiversity Future Center—NBFC”.

## Conflicts of Interest

The authors declare no conflicts of interest.

## Supporting information


**Appendix S1:** ece373906‐sup‐0001‐AppendixS1.xlsx.

**Table S1:** Comparison between three generalized linear mixed models: best model and the same model including spatial autocorrelation or location as random effect.
**Table S2:** Capture locations and morphometric data of lizards included in the analyses.
**Table S3:** Candidate models assessing the role of landscape composition metrics in explaining autotomy frequency.
**Table S4:** Performance of models testing different landscape fragmentation or predator related metrics as additional predictors of caudal autotomy.
**Table S5:** List of the vertebrate predator species occurring in Italy and potentially preying upon *Podarcis muralis* and *Podarcis siculus*.

## Data Availability

The data used in the manuscript are available in Appendix S1.
