# Supplementary material for: A Tale of Tail Loss: Fine‐Scale Landscape Composition Predicts Caudal Autotomy Along an Urban Gradient in Two Congeneric Lizards
Source: Ecol Evol. 2026 Jun 28;16(7):e73906. doi: 10.1002/ece3.73906 (PMC13310963; doi:10.1002/ece3.73906)
Supplement: Supplementary file 2 — Table S1: Comparison between three generalized linear mixed models: best model and the same model including spatial autocorrelation or location as random effect. Table S2: Capture locations and morphometric data of lizards included in the analyses. Table S3: Candidate models assessing the role of landscape composition metrics in explaining autotomy frequency. Table S4: Performance of models testing different landscape fragmentation or predator related metrics as additional predictors of caudal autotomy. Table S5: List of the vertebrate predator species occurring in Italy and potentially preying upon Podarcis muralis and Podarcis siculus. [file ECE3-16-e73906-s002.pdf]

Supplementary information for:

A tale of tail loss: fine-scale landscape composition predicts caudal autotomy along an urban gradient in two congeneric lizards

Natan Gottari, Mattia Falaschi, Stéphanie Sherpa, Agostino Apa, Giulia Arioli, Klevisa Bezhani, Bruno D'Adda, Gentile Francesco Ficetola, Chiara Marialuisa Manzo, Francesco Rosadini, Andrea Melotto

Table of contents:

- **Table S1.** Comparison between three generalized linear mixed models: best model and the same model including spatial autocorrelation or location as random effect.
- **Table S2.** Capture locations and morphometric data of lizards included in the analyses.
- **Table S3.** Candidate models assessing the role of landscape composition metrics in explaining autotomy frequency.
- **Table S4.** Performance of models testing different landscape fragmentation or predator-related metrics as additional predictors of caudal autotomy.
- **Table S5.** List of the vertebrate predator species occurring in Italy and potentially preying upon *Podarcis muralis* and *Podarcis siculus*.

Table S1. Comparison between three generalized linear mixed models (GLMMs): best model and the same model including spatial autocorrelation or location as random effect. This comparison was used to assess whether the results of the final model were affected by the non-independence of lizards captured in the same locality, or by spatial autocorrelation. Model (1) is the final GLMM identified as the best-supported model. Model (2) extends the same GLMM by including "locality" as an additional random effect to account for potential non-independence of individuals sampled within the same site. Model (3) accounts for spatial autocorrelation using a penalized quasi-likelihood approach (glmmPQL), including a spatial correlation structure based on the geographic coordinates of sampling points. All models include the same fixed effects. The significance values (p) of the terms in the three models are shown. The close similarity among values of the three models indicates that neither locality-level clustering nor spatial autocorrelation substantially bias the results of the final GLMM.

Model	Formula	p			
		sex	SVL	species	forest50
1	glmer(regeneration ~ sex + SVL + species + forest50 + (1|city), family=binomial)	0.741	0.002	0.015	0.006
2	glmer(regeneration ~ sex + SVL + species + forest50 + (1|city) + (1|locality), family=binomial)	0.741	<0.001	0.015	0.006
3	glmmPQL(regeneration ~ sex + SVL + species + forest50 + (1|city), correlation=corGaus(form=~lat+long), family=binomial)	0.740	<0.001	0.013	0.005

Table S2 Capture locations and morphometric data of lizards included in the analyses. The table shows: species identity, sampling location (city and coordinates), sex, snout-vent length (SVL), and tail regeneration status (1 = regenerated tail; 0 = intact tail).

Individual_ID	Species	City	Latitude	Longitude	Sex	SVL (mm)	Regeneration
PIM_001	*Podarcis muralis*	Milan	45.4732789	9.1961290	F	66	1
PIM_003	*Podarcis muralis*	Milan	45.4735730	9.1960940	F	52	1
PIM_004	*Podarcis muralis*	Milan	45.4744340	9.1969160	F	52	1
PIM_006	*Podarcis muralis*	Milan	45.4751990	9.1971000	F	57	1
PIM_007	*Podarcis muralis*	Milan	45.4751980	9.1970900	F	55	1
CAS_011	*Podarcis muralis*	Milan	45.4696970	9.1806389	F	64	1
CAS_012	*Podarcis muralis*	Milan	45.4696969	9.1806389	F	67	1
CAS_014	*Podarcis muralis*	Milan	45.4697520	9.1805718	F	61	1
PIM_024	*Podarcis muralis*	Milan	45.4747029	9.2025654	F	66	1
PIM_025	*Podarcis muralis*	Milan	45.4747120	9.2024254	F	57	1
PIM_026	*Podarcis muralis*	Milan	45.4747808	9.2024924	F	59	1
PIM_028	*Podarcis muralis*	Milan	45.4747807	9.2024924	F	63	0
BIC_036	*Podarcis muralis*	Milan	45.5132476	9.2094851	F	61	1
BIC_037	*Podarcis muralis*	Milan	45.5114148	9.2087618	F	54	1
BIC_039	*Podarcis muralis*	Milan	45.5112660	9.2086630	F	60	1
BIC_041	*Podarcis muralis*	Milan	45.5136891	9.2098535	F	55	1
BIC_042	*Podarcis muralis*	Milan	45.5136576	9.2099629	F	69	1
LMB_051	*Podarcis muralis*	Milan	45.4994213	9.2480225	F	55	0
LMB_052	*Podarcis muralis*	Milan	45.4993946	9.2482492	F	65	0
COR_053	*Podarcis muralis*	Milan	45.4584660	9.4876310	F	60	1
COR_056	*Podarcis muralis*	Milan	45.4617110	9.4902400	F	62	0
COR_058	*Podarcis muralis*	Milan	45.4618820	9.4899680	F	55	0
COR_061	*Podarcis muralis*	Milan	45.4617980	9.4900510	F	60	0
NAV_065	*Podarcis muralis*	Milan	45.4483520	9.1713940	F	56	1
NAV_066	*Podarcis muralis*	Milan	45.4482730	9.1713380	F	53	1
NAV_070	*Podarcis muralis*	Milan	45.4479430	9.1709550	F	57	1
NAV_073	*Podarcis muralis*	Milan	45.4508530	9.1708240	F	63	1
NAV_074	*Podarcis muralis*	Milan	45.4500530	9.1700680	F	63	1
NAV_075	*Podarcis muralis*	Milan	45.4492860	9.1708220	F	69	1
LMB_077	*Podarcis muralis*	Milan	45.4953270	9.2476970	F	59	1
LMB_078	*Podarcis muralis*	Milan	45.4953260	9.2476960	F	62	1
LMB_079	*Podarcis muralis*	Milan	45.4951700	9.2473710	F	56	1
PNO_084	*Podarcis muralis*	Milan	45.5324285	9.1763041	F	59	1
PNO_089	*Podarcis muralis*	Milan	45.5352900	9.1789510	F	63	1
PNO_090	*Podarcis muralis*	Milan	45.5352940	9.1789550	F	54	0
PNO_091	*Podarcis muralis*	Milan	45.5352930	9.1789540	F	63	1
FAG_102	*Podarcis muralis*	Milan	45.4334890	8.8294080	F	57	1
FAG_104	*Podarcis muralis*	Milan	45.4321580	8.8298200	F	61	1
FAG_108	*Podarcis muralis*	Milan	45.4289340	8.8270670	F	66	1

FAG_110	*Podarcis muralis*	Milan	45.4307010	8.8291460	F	65	1
GRO_111	*Podarcis muralis*	Milan	45.5754740	9.0931340	F	61	1
GRO_112	*Podarcis muralis*	Milan	45.5755330	9.0932200	F	55	1
GRO_113	*Podarcis muralis*	Milan	45.5755020	9.0929390	F	66	1
GRO_123	*Podarcis muralis*	Milan	45.5788200	9.0965280	F	55	1
MON_124	*Podarcis siculus*	Campobasso	41.5624390	14.6566750	F	56	1
MON_131	*Podarcis siculus*	Campobasso	41.5624010	14.6568580	F	55	1
MON_133	*Podarcis siculus*	Campobasso	41.5634250	14.6550170	F	54	0
UMO_138	*Podarcis siculus*	Campobasso	41.5531748	14.6687079	F	52	0
UMO_139	*Podarcis siculus*	Campobasso	41.5539190	14.6680025	F	64	0
UMO_141	*Podarcis siculus*	Campobasso	41.5540158	14.6684987	F	63	1
UMO_142	*Podarcis siculus*	Campobasso	41.5545738	14.6674097	F	63	1
UMO_143	*Podarcis siculus*	Campobasso	41.5548950	14.6668590	F	61	0
CSA_144	*Podarcis siculus*	Campobasso	41.5885810	14.6905520	F	69	0
CSA_145	*Podarcis siculus*	Campobasso	41.5888170	14.6923010	F	63	0
CSA_146	*Podarcis siculus*	Campobasso	41.5886630	14.6905890	F	62	0
CSA_147	*Podarcis siculus*	Campobasso	41.5873170	14.6950750	F	67	1
CSA_151	*Podarcis siculus*	Campobasso	41.5866550	14.6936580	F	63	1
ORA_154	*Podarcis siculus*	Campobasso	41.5871040	14.5840480	F	54	0
ORA_156	*Podarcis siculus*	Campobasso	41.5864900	14.5833340	F	58	0
ORA_160	*Podarcis siculus*	Campobasso	41.5864950	14.5833350	F	62	1
HOS_163	*Podarcis siculus*	Campobasso	41.5483560	14.6170290	F	66	1
HOS_165	*Podarcis siculus*	Campobasso	41.5492810	14.6122610	F	58	0
TRA_167	*Podarcis siculus*	Campobasso	41.5393990	14.6723220	F	69	1
TRA_168	*Podarcis siculus*	Campobasso	41.5394990	14.6723430	F	64	1
TRA_169	*Podarcis siculus*	Campobasso	41.5394160	14.6724850	F	67	1
TRA_172	*Podarcis siculus*	Campobasso	41.5395640	14.6723600	F	67	1
TRA_173	*Podarcis siculus*	Campobasso	41.5414420	14.6699890	F	55	1
TRA_174	*Podarcis siculus*	Campobasso	41.5411750	14.6707870	F	61	0
PIM_802	*Podarcis muralis*	Milan	45.4746890	9.2023370	F	59	0
PIM_807	*Podarcis muralis*	Milan	45.4749166	9.1997772	F	61	1
PIM_808	*Podarcis muralis*	Milan	45.4750312	9.1997948	F	59	0
PIM_809	*Podarcis muralis*	Milan	45.4749610	9.1998250	F	61	1
PIM_810	*Podarcis muralis*	Milan	45.4750038	9.1997389	F	61	0
PIM_814	*Podarcis muralis*	Milan	45.4749330	9.1996540	F	65	1
SBA_212	*Podarcis muralis*	Florence	43.7881620	11.3234170	F	52	0
SBA_203	*Podarcis muralis*	Florence	43.7893550	11.3196800	F	61	1
SBA_205	*Podarcis muralis*	Florence	43.7889180	11.3192900	F	54	1
SBA_201	*Podarcis siculus*	Florence	43.7918550	11.3198680	F	62	0
SBA_202	*Podarcis siculus*	Florence	43.7904190	11.3194850	F	72	0
SBA_208	*Podarcis siculus*	Florence	43.7889310	11.3226950	F	65	1
SBA_215	*Podarcis siculus*	Florence	43.7928930	11.3208520	F	57	0
ARG_216	*Podarcis siculus*	Florence	43.7837660	11.1958000	F	72	1
ARG_217	*Podarcis siculus*	Florence	43.7867640	11.1970350	F	68	1
ARG_219	*Podarcis siculus*	Florence	43.7845189	11.1957211	F	65	0
ARG_220	*Podarcis siculus*	Florence	43.7850420	11.1955350	F	63	1

ARG_221	*Podarcis siculus*	Florence	43.7846510	11.1953320	F	60	0
ARG_224	*Podarcis siculus*	Florence	43.7865890	11.2012210	F	65	1
ARG_225	*Podarcis muralis*	Florence	43.7858620	11.2006760	F	65	1
SMS_231	*Podarcis siculus*	Florence	43.7767120	11.2357160	F	67	1
SMS_235	*Podarcis siculus*	Florence	43.7767170	11.2358590	F	68	1
SMS_236	*Podarcis siculus*	Florence	43.7769420	11.2349380	F	68	1
SMS_238	*Podarcis muralis*	Florence	43.7773180	11.2365580	F	64	0
SMS_239	*Podarcis muralis*	Florence	43.7773490	11.2365400	F	63	1
SMS_241	*Podarcis muralis*	Florence	43.7773300	11.2365550	F	66	1
SMS_244	*Podarcis muralis*	Florence	43.7774370	11.2364100	F	60	1
CAP_245	*Podarcis siculus*	Florence	43.7651770	11.2735560	F	69	1
CAP_246	*Podarcis siculus*	Florence	43.7651370	11.2744220	F	66	0
CAP_250	*Podarcis siculus*	Florence	43.7653260	11.2745200	F	60	1
CAP_251	*Podarcis siculus*	Florence	43.7650880	11.2757200	F	60	0
CAP_252	*Podarcis siculus*	Florence	43.7655290	11.2744510	F	61	1
CAP_254	*Podarcis siculus*	Florence	43.7653090	11.2757740	F	58	1
CAP_255	*Podarcis muralis*	Florence	43.7652470	11.2730570	F	58	1
CAP_258	*Podarcis muralis*	Florence	43.7654920	11.2730140	F	58	1
CAP_259	*Podarcis muralis*	Florence	43.7653440	11.2730280	F	56	1
CAP_263	*Podarcis muralis*	Florence	43.7654930	11.2730150	F	57	0
CAP_265	*Podarcis muralis*	Florence	43.7654550	11.2726120	F	56	0
CAP_266	*Podarcis muralis*	Florence	43.7653550	11.2730420	F	56	1
AZE_268	*Podarcis muralis*	Florence	43.7737910	11.2672220	F	51	1
AZE_269	*Podarcis muralis*	Florence	43.7735860	11.2674680	F	63	1
AZE_272	*Podarcis muralis*	Florence	43.7736380	11.2678690	F	59	1
AZE_273	*Podarcis muralis*	Florence	43.7738073	11.2668175	F	61	0
AZE_274	*Podarcis muralis*	Florence	43.7736250	11.2678650	F	66	1
AZE_275	*Podarcis muralis*	Florence	43.7735240	11.2676100	F	50	0
AZE_276	*Podarcis muralis*	Florence	43.7735940	11.2678860	F	59	0
GIR_278	*Podarcis siculus*	Florence	43.7628280	11.2630730	F	52	1
GIR_280	*Podarcis siculus*	Florence	43.7627350	11.2627560	F	59	1
GIR_281	*Podarcis siculus*	Florence	43.7628640	11.2631920	F	63	1
GIR_282	*Podarcis siculus*	Florence	43.7629070	11.2631630	F	65	0
GIR_284	*Podarcis siculus*	Florence	43.7626770	11.2631270	F	69	1
GIR_285	*Podarcis siculus*	Florence	43.7628147	11.2628260	F	61	0
GIR_288	*Podarcis siculus*	Florence	43.7627680	11.2630210	F	62	1
GIR_289	*Podarcis siculus*	Florence	43.7626619	11.2628109	F	66	1
LEO_294	*Podarcis muralis*	Florence	43.7597530	11.2549930	F	62	1
LEO_295	*Podarcis muralis*	Florence	43.7594720	11.2549920	F	63	1
LEO_298	*Podarcis muralis*	Florence	43.7579730	11.2528850	F	62	1
LEO_300	*Podarcis muralis*	Florence	43.7574680	11.2482400	F	51	0
LEO_301	*Podarcis muralis*	Florence	43.7581110	11.2482800	F	51	0
PIA_302	*Podarcis muralis*	Florence	43.8243107	11.1730722	F	62	0
PIA_309	*Podarcis muralis*	Florence	43.8246400	11.1733330	F	54	1
PIA_313	*Podarcis muralis*	Florence	43.8249290	11.1734670	F	64	0
PIA_314	*Podarcis siculus*	Florence	43.8240590	11.1728640	F	69	1

PIA_318	*Podarcis siculus*	Florence	43.8243790	11.1732050	F	67	1
PIA_319	*Podarcis siculus*	Florence	43.8247150	11.1734260	F	63	1
THY_322	*Podarcis muralis*	Turin	45.0889435	7.6372485	F	51	0
THY_324	*Podarcis muralis*	Turin	45.0909164	7.6390982	F	65	0
THY_329	*Podarcis muralis*	Turin	45.0903864	7.6386617	F	53	0
THY_330	*Podarcis muralis*	Turin	45.0890623	7.6384448	F	60	1
THY_331	*Podarcis muralis*	Turin	45.0888690	7.6378070	F	63	1
MAN_338	*Podarcis muralis*	Turin	45.0698636	7.7813492	F	64	0
MAN_339	*Podarcis muralis*	Turin	45.0698637	7.7813493	F	60	1
MAN_343	*Podarcis muralis*	Turin	45.0698638	7.7813494	F	64	0
MAN_344	*Podarcis muralis*	Turin	45.0698639	7.7813495	F	64	0
MAN_348	*Podarcis muralis*	Turin	45.0698640	7.7813496	F	61	0
DFR_356	*Podarcis muralis*	Turin	45.0712397	7.6498593	F	62	0
DFR_360	*Podarcis muralis*	Turin	45.0711940	7.6498620	F	67	1
DFR_362	*Podarcis muralis*	Turin	45.0712380	7.6498490	F	64	1
DFR_363	*Podarcis muralis*	Turin	45.0717870	7.6496540	F	58	1
POR_380	*Podarcis muralis*	Turin	45.0651240	7.5766170	F	65.5	1
POR_378	*Podarcis muralis*	Turin	45.0652380	7.5767080	F	63.5	1
POR_379	*Podarcis muralis*	Turin	45.0651230	7.5766160	F	60	0
MUS_367	*Podarcis muralis*	Turin	45.1165220	7.4812760	F	55	0
MUS_388	*Podarcis muralis*	Turin	45.1157570	7.4752490	F	53	1
MUS_389	*Podarcis muralis*	Turin	45.1158125	7.4752783	F	58	0
MUS_394	*Podarcis muralis*	Turin	45.1153490	7.4788440	F	63	1
MUS_391	*Podarcis muralis*	Turin	45.1177790	7.4771670	F	63	0
MUS_392	*Podarcis muralis*	Turin	45.1203870	7.4793860	F	60	0
VAL_400	*Podarcis muralis*	Turin	44.9950810	7.4601340	F	51	0
VAL_405	*Podarcis muralis*	Turin	44.9972050	7.4584480	F	59	0
VAL_409	*Podarcis muralis*	Turin	44.9967720	7.4580960	F	51	1
VAL_410	*Podarcis muralis*	Turin	44.9967400	7.4580930	F	58	1
BUS_415	*Podarcis muralis*	Turin	45.0753240	7.7021150	F	64	0
BUS_419	*Podarcis muralis*	Turin	45.0741450	7.6976890	F	52	0
BUS_420	*Podarcis muralis*	Turin	45.0745800	7.6984710	F	58	1
PDV_441	*Podarcis muralis*	Turin	45.0570257	7.6898883	F	55	1
PDV_447	*Podarcis muralis*	Turin	45.0560838	7.6892197	F	62	1
PDV_450	*Podarcis muralis*	Turin	45.0529260	7.6861590	F	61	0
PDV_451	*Podarcis muralis*	Turin	45.0509239	7.6850511	F	62	0
LIN_462	*Podarcis muralis*	Turin	45.0368150	7.6660090	F	55	1
LIN_463	*Podarcis muralis*	Turin	45.0348160	7.6679860	F	63.5	0
LIN_466	*Podarcis muralis*	Turin	45.0368920	7.6660360	F	66	0
LIN_467	*Podarcis muralis*	Turin	45.0357056	7.6650178	F	55	1
GUG_472	*Podarcis muralis*	Turin	45.0706582	7.6736055	F	64	1
GUG_480	*Podarcis muralis*	Turin	45.0711057	7.6728445	F	58	1
GUG_482	*Podarcis muralis*	Turin	45.0713790	7.6731860	F	53.5	1
GUG_483	*Podarcis muralis*	Turin	45.0712910	7.6732440	F	60	0
PIM_002	*Podarcis muralis*	Milan	45.4734810	9.1960380	M	65	1
PIM_005	*Podarcis muralis*	Milan	45.4751970	9.1970800	M	66	1

CAS_010	*Podarcis muralis*	Milan	45.4696970	9.1806389	M	66	1
CAS_013	*Podarcis muralis*	Milan	45.4697436	9.1806640	M	66	1
CAS_015	*Podarcis muralis*	Milan	45.4696969	9.1806388	M	68	1
CAS_016	*Podarcis muralis*	Milan	45.4697500	9.1805816	M	65	1
CAS_017	*Podarcis muralis*	Milan	45.4697306	9.1804233	M	67	1
CAS_021	*Podarcis muralis*	Milan	45.4717740	9.1779207	M	59	1
CAS_022	*Podarcis muralis*	Milan	45.4717402	9.1778490	M	62	1
PIM_023	*Podarcis muralis*	Milan	45.4747621	9.2024564	M	61	0
PIM_030	*Podarcis muralis*	Milan	45.4748465	9.2024143	M	70	1
PIM_033	*Podarcis muralis*	Milan	45.4754892	9.1990141	M	70	1
PIM_034	*Podarcis muralis*	Milan	45.4750992	9.1989667	M	66	0
PIM_035	*Podarcis muralis*	Milan	45.4749540	9.1999097	M	63	1
BIC_038	*Podarcis muralis*	Milan	45.5114862	9.2088101	M	64	1
BIC_040	*Podarcis muralis*	Milan	45.5116798	9.2087645	M	52	0
BIC_043	*Podarcis muralis*	Milan	45.5136575	9.2099628	M	53	1
BIC_044	*Podarcis muralis*	Milan	45.5133685	9.2092846	M	63	0
BIC_045	*Podarcis muralis*	Milan	45.5130290	9.2088002	M	64	1
BIC_046	*Podarcis muralis*	Milan	45.5131330	9.2091140	M	69	1
LMB_047	*Podarcis muralis*	Milan	45.4993781	9.2479823	M	60	0
LMB_049	*Podarcis muralis*	Milan	45.4994580	9.2481754	M	63	1
LMB_050	*Podarcis muralis*	Milan	45.4994773	9.2480145	M	55	0
COR_054	*Podarcis muralis*	Milan	45.4594810	9.4911450	M	55	1
COR_055	*Podarcis muralis*	Milan	45.4597440	9.4927150	M	72	1
COR_057	*Podarcis muralis*	Milan	45.4618310	9.4899050	M	61	1
COR_059	*Podarcis muralis*	Milan	45.4617990	9.4900520	M	61	1
COR_060	*Podarcis muralis*	Milan	45.4617960	9.4900490	M	61	0
COR_062	*Podarcis muralis*	Milan	45.4628350	9.4876820	M	64	1
COR_063	*Podarcis muralis*	Milan	45.4601095	9.4904097	M	56	1
NAV_067	*Podarcis muralis*	Milan	45.4478470	9.1709320	M	58	1
NAV_068	*Podarcis muralis*	Milan	45.4484410	9.1714990	M	65	1
NAV_069	*Podarcis muralis*	Milan	45.4483990	9.1714170	M	56	1
NAV_071	*Podarcis muralis*	Milan	45.4498740	9.1747210	M	50	0
NAV_072	*Podarcis muralis*	Milan	45.4508640	9.1708140	M	65	0
LMB_076	*Podarcis muralis*	Milan	45.4953250	9.2476950	M	55	1
LMB_080	*Podarcis muralis*	Milan	45.4949430	9.2477360	M	61	1
LMB_081	*Podarcis muralis*	Milan	45.4993820	9.2479450	M	64	1
LMB_082	*Podarcis muralis*	Milan	45.4994560	9.2480800	M	61	0
PNO_083	*Podarcis muralis*	Milan	45.5324350	9.1759850	M	65	1
PNO_085	*Podarcis muralis*	Milan	45.5330150	9.1765500	M	65	1
PNO_086	*Podarcis muralis*	Milan	45.5337060	9.1772240	M	70	1
PNO_087	*Podarcis muralis*	Milan	45.5317760	9.1762150	M	58	1
PNO_088	*Podarcis muralis*	Milan	45.5348780	9.1785150	M	60	0
PNO_092	*Podarcis muralis*	Milan	45.5352920	9.1789530	M	61	1
FAG_100	*Podarcis muralis*	Milan	45.4338800	8.8326190	M	71	0
FAG_101	*Podarcis muralis*	Milan	45.4342310	8.8314830	M	68	1
FAG_103	*Podarcis muralis*	Milan	45.4334000	8.8293490	M	65	1

FAG_105	*Podarcis muralis*	Milan	45.4321390	8.8291560	M	58	0
FAG_106	*Podarcis muralis*	Milan	45.4319680	8.8279430	M	70	1
FAG_107	*Podarcis muralis*	Milan	45.4287840	8.8269590	M	72	1
FAG_109	*Podarcis muralis*	Milan	45.4293580	8.8278700	M	71	0
GRO_114	*Podarcis muralis*	Milan	45.5759970	9.0934840	M	60	1
GRO_115	*Podarcis muralis*	Milan	45.5755170	9.0930570	M	66	1
GRO_116	*Podarcis muralis*	Milan	45.5754900	9.0929800	M	56	1
GRO_117	*Podarcis muralis*	Milan	45.5759940	9.0935290	M	63	1
GRO_118	*Podarcis muralis*	Milan	45.5760380	9.0922640	M	60	1
GRO_119	*Podarcis muralis*	Milan	45.5759820	9.0933300	M	69	1
GRO_120	*Podarcis muralis*	Milan	45.5773800	9.0946890	M	71	1
GRO_121	*Podarcis muralis*	Milan	45.5788270	9.0952080	M	65	0
GRO_122	*Podarcis muralis*	Milan	45.5787920	9.0965710	M	65	1
MON_125	*Podarcis siculus*	Campobasso	41.5626276	14.6559920	M	58	0
MON_126	*Podarcis siculus*	Campobasso	41.5627420	14.6560320	M	63	0
MON_127	*Podarcis siculus*	Campobasso	41.5625650	14.6569000	M	60	0
MON_128	*Podarcis siculus*	Campobasso	41.5625640	14.6568900	M	62	1
MON_129	*Podarcis siculus*	Campobasso	41.5624360	14.6567490	M	70	1
MON_130	*Podarcis siculus*	Campobasso	41.5625130	14.6564720	M	64	0
UMO_134	*Podarcis siculus*	Campobasso	41.5531748	14.6687079	M	69	1
UMO_136	*Podarcis siculus*	Campobasso	41.5535516	14.6683726	M	70	0
UMO_137	*Podarcis siculus*	Campobasso	41.5532771	14.6686113	M	72	1
UMO_140	*Podarcis siculus*	Campobasso	41.5539250	14.6685570	M	67	1
CSA_148	*Podarcis siculus*	Campobasso	41.5872680	14.6951660	M	65	1
CSA_149	*Podarcis siculus*	Campobasso	41.5873020	14.6950250	M	66	0
CSA_150	*Podarcis siculus*	Campobasso	41.5873040	14.6951090	M	66	1
CSA_152	*Podarcis siculus*	Campobasso	41.5870800	14.6955360	M	61	0
CSA_153	*Podarcis siculus*	Campobasso	41.5871120	14.6954680	M	70	1
ORA_155	*Podarcis siculus*	Campobasso	41.5866240	14.5832990	M	74	1
ORA_157	*Podarcis siculus*	Campobasso	41.5865860	14.5833650	M	73	1
ORA_158	*Podarcis siculus*	Campobasso	41.5865130	14.5833160	M	72	0
ORA_159	*Podarcis siculus*	Campobasso	41.5865760	14.5833070	M	59	0
ORA_161	*Podarcis siculus*	Campobasso	41.5864940	14.5833340	M	62	1
ORA_162	*Podarcis siculus*	Campobasso	41.5871030	14.5840470	M	68	1
HOS_164	*Podarcis siculus*	Campobasso	41.5492290	14.6126940	M	69	1
TRA_166	*Podarcis siculus*	Campobasso	41.5393980	14.6723210	M	75	1
TRA_170	*Podarcis siculus*	Campobasso	41.5394000	14.6725290	M	70	1
TRA_171	*Podarcis siculus*	Campobasso	41.5394990	14.6724340	M	62	0
TRA_175	*Podarcis siculus*	Campobasso	41.5413660	14.6771006	M	60	0
HOS_176	*Podarcis siculus*	Campobasso	41.5486780	14.6117330	M	62	1
PIM_801	*Podarcis muralis*	Milan	45.4747165	9.2024172	M	55	0
PIM_804	*Podarcis muralis*	Milan	45.4747033	9.2024510	M	63	1
PIM_805	*Podarcis muralis*	Milan	45.4747200	9.2023470	M	65	1
PIM_806	*Podarcis muralis*	Milan	45.4749860	9.1998010	M	64	1
SBA_206	*Podarcis muralis*	Florence	43.7894050	11.3219800	M	52	0
SBA_211	*Podarcis muralis*	Florence	43.7881170	11.3235470	M	64	1

SBA_207	*Podarcis muralis*	Florence	43.7894710	11.3222440	M	55	1
SBA_213	*Podarcis muralis*	Florence	43.7889600	11.3228170	M	55	0
SBA_204	*Podarcis siculus*	Florence	43.7893230	11.3191200	M	73	1
SBA_209	*Podarcis siculus*	Florence	43.7885820	11.3228910	M	67	0
SBA_214	*Podarcis siculus*	Florence	43.7897190	11.3196750	M	66	0
ARG_218	*Podarcis siculus*	Florence	43.7860090	11.2009430	M	74	1
ARG_222	*Podarcis siculus*	Florence	43.7846510	11.1959430	M	70	1
ARG_223	*Podarcis siculus*	Florence	43.7846780	11.1953380	M	78	1
SMS_229	*Podarcis siculus*	Florence	43.7776720	11.2372030	M	74	0
SMS_230	*Podarcis siculus*	Florence	43.7770090	11.2367460	M	74	1
SMS_232	*Podarcis siculus*	Florence	43.7766620	11.2343630	M	71	1
SMS_233	*Podarcis siculus*	Florence	43.7767440	11.2344710	M	64	0
SMS_234	*Podarcis siculus*	Florence	43.7768760	11.2354660	M	67	0
SMS_237	*Podarcis siculus*	Florence	43.7766440	11.2359040	M	62	1
SMS_227	*Podarcis muralis*	Florence	43.7775450	11.2361550	M	46	1
SMS_228	*Podarcis muralis*	Florence	43.7775440	11.2361540	M	61	1
SMS_240	*Podarcis muralis*	Florence	43.7773650	11.2364940	M	72	1
SMS_242	*Podarcis muralis*	Florence	43.7773980	11.2364340	M	67	1
SMS_243	*Podarcis muralis*	Florence	43.7774360	11.2364000	M	68	1
CAP_247	*Podarcis siculus*	Florence	43.7653120	11.2723090	M	63	1
CAP_248	*Podarcis siculus*	Florence	43.7651500	11.2755720	M	67	1
CAP_249	*Podarcis siculus*	Florence	43.7651220	11.2756760	M	64	0
CAP_253	*Podarcis siculus*	Florence	43.7651210	11.2756790	M	71	0
CAP_260	*Podarcis siculus*	Florence	43.7655350	11.2734590	M	65	1
CAP_267	*Podarcis siculus*	Florence	43.7654110	11.2732260	M	70	1
CAP_256	*Podarcis muralis*	Florence	43.7653940	11.2730830	M	55	0
CAP_261	*Podarcis muralis*	Florence	43.7653810	11.2730280	M	64	1
CAP_264	*Podarcis muralis*	Florence	43.7655110	11.2730630	M	60	0
AZE_270	*Podarcis muralis*	Florence	43.7737930	11.2672250	M	63	1
AZE_271	*Podarcis muralis*	Florence	43.7737920	11.2672240	M	66	1
AZE_277	*Podarcis muralis*	Florence	43.7737932	11.2678300	M	57	1
GIR_279	*Podarcis siculus*	Florence	43.7626560	11.2629200	M	69	1
GIR_283	*Podarcis siculus*	Florence	43.7628560	11.2628400	M	71	1
GIR_286	*Podarcis siculus*	Florence	43.7627130	11.2626543	M	74	1
GIR_290	*Podarcis siculus*	Florence	43.7628040	11.2625510	M	78	1
GIR_291	*Podarcis siculus*	Florence	43.7629340	11.2628880	M	70	1
LEO_293	*Podarcis muralis*	Florence	43.7602843	11.2550244	M	60	1
LEO_296	*Podarcis muralis*	Florence	43.7588570	11.2540230	M	71	1
LEO_297	*Podarcis muralis*	Florence	43.7580600	11.2513750	M	63	0
LEO_299	*Podarcis muralis*	Florence	43.7574690	11.2482500	M	67	0
LEO_292	*Podarcis muralis*	Florence	43.7658010	11.2555530	M	56	0
PIA_304	*Podarcis muralis*	Florence	43.8241242	11.1729941	M	68	0
PIA_305	*Podarcis muralis*	Florence	43.8248420	11.1734620	M	55	0
PIA_306	*Podarcis muralis*	Florence	43.8243260	11.1730870	M	60	1
PIA_307	*Podarcis muralis*	Florence	43.8241490	11.1729870	M	60	1
PIA_308	*Podarcis muralis*	Florence	43.8240950	11.1729510	M	60	0

PIA_310	*Podarcis muralis*	Florence	43.8241477	11.1730558	M	68	1
PIA_311	*Podarcis muralis*	Florence	43.8244390	11.1728010	M	69	1
PIA_317	*Podarcis muralis*	Florence	43.8240860	11.1729630	M	61	1
PIA_303	*Podarcis siculus*	Florence	43.8241527	11.1730370	M	74	1
PIA_312	*Podarcis siculus*	Florence	43.8242420	11.1731090	M	66	0
PIA_315	*Podarcis siculus*	Florence	43.8242650	11.1732130	M	68	0
PIA_316	*Podarcis siculus*	Florence	43.8244130	11.1731750	M	72	0
THY_320	*Podarcis muralis*	Turin	45.0889326	7.6382758	M	55	1
THY_321	*Podarcis muralis*	Turin	45.0893900	7.6385300	M	63.5	0
THY_323	*Podarcis muralis*	Turin	45.0908520	7.6389700	M	65	1
THY_325	*Podarcis muralis*	Turin	45.0889447	7.6383217	M	65	1
THY_327	*Podarcis muralis*	Turin	45.0890624	7.6384449	M	70	0
THY_332	*Podarcis muralis*	Turin	45.0889293	7.6372261	M	67	1
MAN_335	*Podarcis muralis*	Turin	45.0697780	7.7812220	M	60	1
MAN_336	*Podarcis muralis*	Turin	45.0693400	7.7809530	M	71	1
MAN_337	*Podarcis muralis*	Turin	45.0698638	7.7813499	M	65	1
MAN_340	*Podarcis muralis*	Turin	45.0680330	7.7799680	M	68	1
MAN_346	*Podarcis muralis*	Turin	45.0696970	7.7812590	M	62.5	1
MAN_350	*Podarcis muralis*	Turin	45.0660460	7.7788220	M	58	0
DFR_354	*Podarcis muralis*	Turin	45.0711869	7.6498073	M	65	1
DFR_357	*Podarcis muralis*	Turin	45.0709736	7.6495022	M	54	1
DFR_361	*Podarcis muralis*	Turin	45.0717860	7.6496530	M	62	1
DFR_365	*Podarcis muralis*	Turin	45.0728219	7.6497664	M	63	1
DFR_366	*Podarcis muralis*	Turin	45.0725056	7.6505127	M	62	1
DFR_364	*Podarcis muralis*	Turin	45.0726840	7.6494570	M	64.5	1
DFR_355	*Podarcis muralis*	Turin	45.0712370	7.6498480	M	61	1
DFR_352	*Podarcis muralis*	Turin	45.0716078	7.6493129	M	67	1
POR_370	*Podarcis muralis*	Turin	45.0648080	7.5734807	M	55	1
POR_372	*Podarcis muralis*	Turin	45.0662090	7.5776800	M	67	1
POR_374	*Podarcis muralis*	Turin	45.0655710	7.5754140	M	64	1
POR_375	*Podarcis muralis*	Turin	45.0652390	7.5770280	M	65	1
POR_377	*Podarcis muralis*	Turin	45.0651590	7.5766610	M	65	0
POR_373	*Podarcis muralis*	Turin	45.0653030	7.5748650	M	58	0
MUS_381	*Podarcis muralis*	Turin	45.1155020	7.4800700	M	59	0
MUS_382	*Podarcis muralis*	Turin	45.1145260	7.4789920	M	59	1
MUS_383	*Podarcis muralis*	Turin	45.1156920	7.4752350	M	60	0
MUS_384	*Podarcis muralis*	Turin	45.1159482	7.4755169	M	57	1
MUS_386	*Podarcis muralis*	Turin	45.1161730	7.4759220	M	61	0
MUS_393	*Podarcis muralis*	Turin	45.1204509	7.4793026	M	60	0
VAL_403	*Podarcis muralis*	Turin	44.9955050	7.4583850	M	54	0
VAL_406	*Podarcis muralis*	Turin	44.9966320	7.4579170	M	55	1
VAL_402	*Podarcis muralis*	Turin	44.9962210	7.4594740	M	50	0
VAL_408	*Podarcis muralis*	Turin	44.9951200	7.4601940	M	51	0
VAL_411	*Podarcis muralis*	Turin	44.9967500	7.4580940	M	51	0
BUS_416	*Podarcis muralis*	Turin	45.0753470	7.7020320	M	65	1
BUS_421	*Podarcis muralis*	Turin	45.0745830	7.6984800	M	64	1

BUS_422	*Podarcis muralis*	Turin	45.0779140	7.7003810	M	62	1
BUS_424	*Podarcis muralis*	Turin	45.0782800	7.7000450	M	65	1
BUS_425	*Podarcis muralis*	Turin	45.0780910	7.7002260	M	65	1
BUS_426	*Podarcis muralis*	Turin	45.0786600	7.7006560	M	61.5	0
PDV_440	*Podarcis muralis*	Turin	45.0569440	7.6898340	M	69	0
PDV_442	*Podarcis muralis*	Turin	45.0566230	7.6895500	M	57	0
PDV_443	*Podarcis muralis*	Turin	45.0567340	7.6896880	M	66	1
PDV_444	*Podarcis muralis*	Turin	45.0561080	7.6891614	M	65	1
PDV_446	*Podarcis muralis*	Turin	45.0563560	7.6896210	M	61	0
PDV_453	*Podarcis muralis*	Turin	45.0529060	7.6862060	M	67	1
LIN_454	*Podarcis muralis*	Turin	45.0348020	7.6680080	M	65	1
LIN_457	*Podarcis muralis*	Turin	45.0347998	7.6679819	M	58	0
LIN_459	*Podarcis muralis*	Turin	45.0347710	7.6679520	M	55	1
LIN_460	*Podarcis muralis*	Turin	45.0347532	7.6679410	M	66	1
LIN_465	*Podarcis muralis*	Turin	45.0368720	7.6660350	M	66	1
LIN_468	*Podarcis muralis*	Turin	45.0360510	7.6653200	M	62	0
GUG_471	*Podarcis muralis*	Turin	45.0706583	7.6736056	M	60	0
GUG_473	*Podarcis muralis*	Turin	45.0708370	7.6728130	M	62	1
GUG_474	*Podarcis muralis*	Turin	45.0713740	7.6731890	M	65	1
GUG_475	*Podarcis muralis*	Turin	45.0714140	7.6731930	M	62	1
GUG_476	*Podarcis muralis*	Turin	45.0709500	7.6730640	M	54	1
GUG_478	*Podarcis muralis*	Turin	45.0712940	7.6729581	M	64	0
GUG_479	*Podarcis muralis*	Turin	45.0713890	7.6731630	M	64	1
VAL_404	*Podarcis muralis*	Turin	44.9965670	7.4578860	M	48	1
VAL_401	*Podarcis muralis*	Turin	44.9964710	7.4606450	M	48	1

Table S3. Candidate models assessing the role of landscape composition metrics in explaining autotomy frequency. Models are ranked by increasing AICc. All models include sex, SVL, and species as fixed factors and city as a random effect. ΔAICc values are calculated relative to the model with the lowest AICc, i.e. the one with forest cover within 50 m, identified as the best-supported model. *w* indicates the AIC weight of the models. Models within ΔAICc < 2 were considered to have similar support; among these, the most parsimonious model was retained for the role of selected fixed factors as drivers of autotomy frequency.

Model	Formula	df	logLik	AICc	ΔAICc	*w*
1	**regeneration ~ sex + SVL + species + forest50 + (1|city)**	**6**	**-232.466**	**477.2**	0	0.235
2	regeneration ~ sex + SVL + species + forest100 + (1|city)	6	-233.617	479.5	2.3	0.074
3	regeneration ~ sex + SVL + species + forest150 + (1|city)	6	-233.968	480.2	3	0.052
4	regeneration ~ sex + SVL + species + forest900 + (1|city)	6	-234.059	480.3	3.1	0.050
5	regeneration ~ sex + SVL + species + forest950 + (1|city)	6	-234.046	480.3	3.1	0.050
6	regeneration ~ sex + SVL + species + forest1000 + (1|city)	6	-234.028	480.3	3.1	0.050
7	regeneration ~ sex + SVL + species + forest850 + (1|city)	6	-234.103	480.4	3.2	0.047
8	regeneration ~ sex + SVL + species + forest200 + (1|city)	6	-234.158	480.5	3.3	0.045
9	regeneration ~ sex + SVL + species + forest800 + (1|city)	6	-234.167	480.6	3.4	0.043
10	regeneration ~ sex + SVL + species + forest750 + (1|city)	6	-234.235	480.7	3.5	0.041
11	regeneration ~ sex + SVL + species + forest700 + (1|city)	6	-234.379	481.0	3.8	0.035
12	regeneration ~ sex + SVL + species + forest650 + (1|city)	6	-234.529	481.3	4.1	0.030
13	regeneration ~ sex + SVL + species + forest250 + (1|city)	6	-234.633	481.5	4.3	0.027
14	regeneration ~ sex + SVL + species + forest600 + (1|city)	6	-234.644	481.5	4.3	0.027
15	regeneration ~ sex + SVL + species + forest550 + (1|city)	6	-234.750	481.7	4.5	0.025
16	regeneration ~ sex + SVL + species + forest300 + (1|city)	6	-234.810	481.8	4.6	0.024
17	regeneration ~ sex + SVL + species + forest500 + (1|city)	6	-234.843	481.9	4.7	0.022
18	regeneration ~ sex + SVL + species + forest350 + (1|city)	6	-234.909	482.0	4.8	0.021
19	regeneration ~ sex + SVL + species + forest450 + (1|city)	6	-234.959	482.1	4.9	0.020
20	regeneration ~ sex + SVL + species + forest400 + (1|city)	6	-235.006	482.2	5	0.019
21	regeneration ~ sex + SVL + species + (1|city)	5	-236.229	482.6	5.4	0.016
22	regeneration ~ sex + SVL + species + grass50 + (1|city)	6	-235.226	482.7	5.5	0.015
23	regeneration ~ sex + SVL + species + grass1000 + (1|city)	6	-235.467	483.2	6	0.012
24	regeneration ~ sex + SVL + species + grass500 + (1|city)	6	-235.879	484.0	6.8	0.008
25	regeneration ~ sex + SVL + species + grass250 + (1|city)	6	-236.143	484.5	7.3	0.006
26	regeneration ~ sex + SVL + species + grass100 + (1|city)	6	-236.229	484.7	7.5	0.006

Table S4. Performance of models testing different landscape fragmentation or predator-related metrics as additional predictors of caudal autotomy. All models included species identity, sex, SVL (snout–vent length) and forest cover within 50 m as fixed factors and city as a random effect. Additionally, these models included a landscape fragmentation metric (number of patches), natural predator-related variables, and cat-related metrics. ΔAICc indicates the difference in AICc compared to the best model in Table S1. For all the models, ΔAICc was >0, indicating a loss of fitness compared to the best AICc model. The coefficients and the associated significance refer to the tested predictor. Abbreviations: lsm_c_np50 = number of patches (50 m scale); pred_GBIF = number of predator species based on occurrence data from GBIF; pred_SDM = number of predator species based on species distribution models; n_cats_500m = number of cat colonies within a 500 m buffer; n_cats_300m = number of cat colonies within a 300 m buffer; Closest_Colony = distance from the closest cat colony (m).

Formula	Predictor added	ΔAICc	*β*	p
regeneration ~ sex + SVL + species + forest50 + lsm_c_np50 + (1|city)	lsm_c_np50	2.8	0.056	0.725
regeneration ~ sex + SVL + species + forest50 + predators_GBIF + (1|city)	predators_GBIF	2.4	-0.012	0.484
regeneration ~ sex + SVL + species + forest50 + predators_SDM + (1|city)	predators_SDM	2.8	-0.014	0.674
regeneration ~ sex + SVL + species + forest50 + n_cats_500m + (1|city)	n_cats_500m	0.9	0.098	0.155
regeneration ~ sex + SVL + species + forest50 + Closest_Colony + (1|city)	Closest_Colony	1.8	-0.058	0.618
regeneration ~ sex + SVL + species + forest50 + n_cats_300m + (1|city)	n_cats_300m	2.2	0.107	0.395

Table S5 List of the vertebrate predator species occurring in Italy and potentially preying upon *Podarcis muralis* and *Podarcis siculus*. The list is based on the database compiled by Maiorano et al. (2020). For each species, columns indicate whether at least one occurrence record (GBIF) or a model-predicted presence (SDMs) was detected around the lizard sampling sites (see text).

Species	Class	GBIF	SDMs
Aquila nipalensis	Aves	–	–
Aquila pomarina	Aves	–	–
Asio flammeus	Aves	✓	–
Asio otus	Aves	✓	✓
Athene noctua	Aves	✓	✓
Bubulcus ibis	Aves	✓	✓
Burhinus oedicnemus	Aves	–	✓
Buteo buteo	Aves	✓	✓
Buteo rufinus	Aves	–	✓
Chlamydotis undulata	Aves	–	–
Ciconia ciconia	Aves	✓	✓
Ciconia nigra	Aves	✓	✓
Circaetus gallicus	Aves	✓	✓
Circus pygargus	Aves	–	✓
Coloeus monedula	Aves	✓	–
Coracias garrulus	Aves	✓	✓
Corvus corax	Aves	✓	✓
Corvus corone	Aves	✓	✓
Corvus frugilegus	Aves	✓	✓
Erithacus rubecula	Aves	✓	✓
Falco biarmicus	Aves	–	–
Falco cherrug	Aves	–	–
Falco tinnunculus	Aves	✓	✓
Falco vespertinus	Aves	✓	✓
Francolinus francolinus	Aves	–	–
Grus grus	Aves	✓	–
Ichthyaetus audouinii	Aves	–	–
Ichthyaetus melanocephalus	Aves	✓	✓
Lanius collurio	Aves	✓	✓
Lanius excubitor	Aves	✓	✓
Lanius meridionalis	Aves	–	✓
Lanius senator	Aves	✓	✓
Larus argentatus	Aves	–	✓
Larus armenicus	Aves	–	✓
Larus cachinnans	Aves	✓	✓
Milvus migrans	Aves	✓	✓
Milvus milvus	Aves	✓	✓
Monticola saxatilis	Aves	–	✓
Monticola solitarius	Aves	✓	✓
Numenius arquata	Aves	–	✓
Oenanthe deserti	Aves	–	–
Oenanthe isabellina	Aves	–	✓
Oenanthe leucura	Aves	–	✓
Oenanthe oenanthe	Aves	✓	✓
Otis tarda	Aves	–	✓
Otus scops	Aves	✓	✓

Pastor roseus	Aves	–	✓
Pernis apivorus	Aves	✓	✓
Phasianus colchicus	Aves	✓	✓
Pica pica	Aves	✓	✓
Saxicola torquatus	Aves	✓	✓
Strix aluco	Aves	✓	✓
Sturnus unicolor	Aves	–	✓
Sturnus vulgaris	Aves	✓	✓
Turdus merula	Aves	✓	✓
Turdus philomelos	Aves	✓	✓
Turdus torquatus	Aves	✓	✓
Tyto alba	Aves	✓	✓
Upupa epops	Aves	✓	✓
Anguis fragilis	Reptilia	–	✓
Chamaeleo chamaeleon	Reptilia	–	–
Coronella girondica	Reptilia	✓	✓
Dolichophis caspius	Reptilia	–	✓
Eirenis modestus	Reptilia	–	–
Elaphe quatuorlineata	Reptilia	–	✓
Eryx jaculus	Reptilia	–	–
Hemorrhois hippocrepis	Reptilia	–	–
Hierophis gemonensis	Reptilia	–	✓
Hierophis viridiflavus	Reptilia	✓	✓
Hierophis viridiflavus carbonarius	Reptilia	–	–
Lacerta agilis	Reptilia	–	✓
Lacerta bilineata	Reptilia	✓	✓
Lacerta media	Reptilia	–	✓
Lacerta trilineata	Reptilia	–	✓
Lacerta viridis	Reptilia	–	✓
Macroprotodon cucullatus	Reptilia	–	–
Malpolon monspessulanus	Reptilia	–	✓
Platyceps najadum	Reptilia	–	✓
Pseudopus apodus	Reptilia	–	✓
Telescopus fallax	Reptilia	–	✓
Timon lepidus	Reptilia	–	✓
Vipera ammodytes	Reptilia	–	✓
Vipera aspis	Reptilia	✓	✓
Vipera berus	Reptilia	–	✓
Vipera ursinii	Reptilia	–	✓
Zamenis lineatus	Reptilia	–	✓
Zamenis longissimus	Reptilia	✓	✓
Zamenis situla	Reptilia	–	✓
Atelerix algirus	Mammalia	–	–
Canis aureus	Mammalia	–	✓
Erinaceus europaeus	Mammalia	✓	✓
Erinaceus roumanicus	Mammalia	–	✓
Felis silvestris	Mammalia	–	✓
Genetta genetta	Mammalia	–	✓
Martes foina	Mammalia	–	✓
Martes martes	Mammalia	–	✓
Meles meles	Mammalia	✓	✓
Mustela nivalis	Mammalia	–	✓
Sus scrofa	Mammalia	✓	✓
Vulpes vulpes	Mammalia	✓	✓
